# The use of metformin, sulfonylurea compounds and insulin and the risk of hip fractures in diabetic patients: a systematic review and meta-analysis of observational studies

**DOI:** 10.1186/s12891-023-06493-9

**Published:** 2023-05-09

**Authors:** Mansour Bahardoust, Mohsen Yarali, Ghazaleh Donyadideh, Elham Rahimi, Delaram Naderi, Farshid Monshizadeh Tehrani, Ali Delpisheh

**Affiliations:** 1 Department of Epidemiology, School of Public Health and Safety, Shahid Beheshti University of Medical Sciences, Tehran, Iran; 2 School of Medicine, Kashan University of Medical Sciences, Kashan, Iran; 3 School of Medicine, Mashad University of Medical Sciences, Mashad, Iran; 4 School of Medicine, Iran University of Medical Sciences, Tehran, Iran; 5 Psychiatric Center Ballerup, Copenhagen University Hospital, Copenhagen, Denmark; 6 Safety Promotion and Injury Prevention Research Center, Shahid Beheshti University of Medical Sciences, Tehran, Iran

**Keywords:** Fracture, Hip fracture, Insulin, Metformin, Sulfonylurea, Anti-diabetic drugs

## Abstract

**Background:**

Hip fracture is a major health problem that occurs more often in the elderly, especially in diabetic patients. Some studies have been conducted regarding the effect of anti- diabetic drugs on fractures. But so far, no meta-analysis study has been conducted to investigate the effect of diabetic drugs on hip fractures. Therefore, this study investigated the relationship between anti-diabetic drugs (Metformin, Sulfonylurea, and insulin) with hip fractures.

**Methods:**

In this systematic review and meta analysis study, PubMed, Scopus, Google Scholar, and Web of Science databases were searched with specific keywords to find relevant studies. Two researchers included related studies after screening based on the title and full text. Cochran’s Q and I2 tests were used to assess heterogeneity between studies. Publication bias between studies was evaluated for each drug using Egger’s test. A 95% confidence interval was used for effect size significance. Overall, 49 studies, including 6,631,297 participants, were reviewed.

**Results:**

The results showed that metformin significantly reduced the risk of hip fracture (HR: 0.833, 95% CI: 0.759, 0.914, P:0.001). Consumption of sulfonylurea compounds was significantly associated with an increased risk of hip fracture. (HR: 1.175, 95% CI:1.068,1.293, P:0.001), The risk of hip fracture in patients receiving insulin was significantly higher than in diabetic patients who did not receive insulin. (HR:1.366, 95% CI:1.226,1.522, P:0.001).

**Conclusion:**

The results of this study showed that taking metformin reduces the risk of hip fracture, and insulin and Sulfonylurea increase the risk of hip fracture.

## Background

Recently, with the aging of the population, the increasing incidence of hip fractures has become a major health problem. It is estimated that by the middle of this century, more than 6 million people will suffer hip fractures yearly, including predominantly elderly people [1]. Hip fracture is one of the main causes of morbidity (30-50% of patients become disabled and lose their functional independence) and mortality (approximately 22% annual mortality).This fracture is one of the most serious consequences of osteoporosis. The worldwide incidence of these fractures is estimated to increase in the coming years worldwide and especially in developing countries in elderly patients, which could lead to increased clinical burden, increased hospitalizations, and related outcomes [2]. These fractures impose a heavy burden on health systems and make the hospital management of these patients a challenge. Ceolin C et al., showed that the functional ability of the elderly hospitalized due to proximal femur fractures decreases significantly in the first 6 months after discharge, which leads to an increased risk of death in these patients in the first year after discharge from the hospital [3]. There are differences in the incidence of hip fractures in people aged 50 years and older in different countries, ranging from an age-standardized rate of more than 500 per 100,000 in Denmark to less than 100 per 100,000 in South Africa [4]. Hip fracture makes the physical conditions of elderly people more complicated despite their diseases. And it also puts a lot of pressure on the healthcare system. Due to the many problems, disease burden, and death rate caused, hip fracture is recognized as the last fracture in life [5, 6]. In the studies conducted so far, the risk factors of hip fracture in old age are sex, smoking, older age, alcohol consumption, blood pressure, diabetes, and osteoporosis [1].

In recent years, it has been almost accepted that diabetes (both T1D and T2D) has major effects on bone metabolism and its fracture, which is generally known as the neglected complications of diabetes. [7]. In recent years, various oral and injectable drugs have been used to treat type 2 diabetes, considering that the risk of fracture is higher in diabetic people [7].

Some studies have been conducted regarding the relationship between various diabetic medications and bone fractures, and there is an inconsistency between the results of these studies. For example, the effect of metformin on bone fractures has been described as reducing in some studies [8–10] and others as ineffective [11, 12]. Based on our knowledge, a meta-analysis has not been conducted to investigate the effect of metformin, insulin, and sulfonylureas on hip fracture. Therefore, this study investigated the effect of three anti-diabetic drugs, metformin, insulin, and sulfonylureas, on hip fractures in patients with diabetes.

**Fig. 1:**
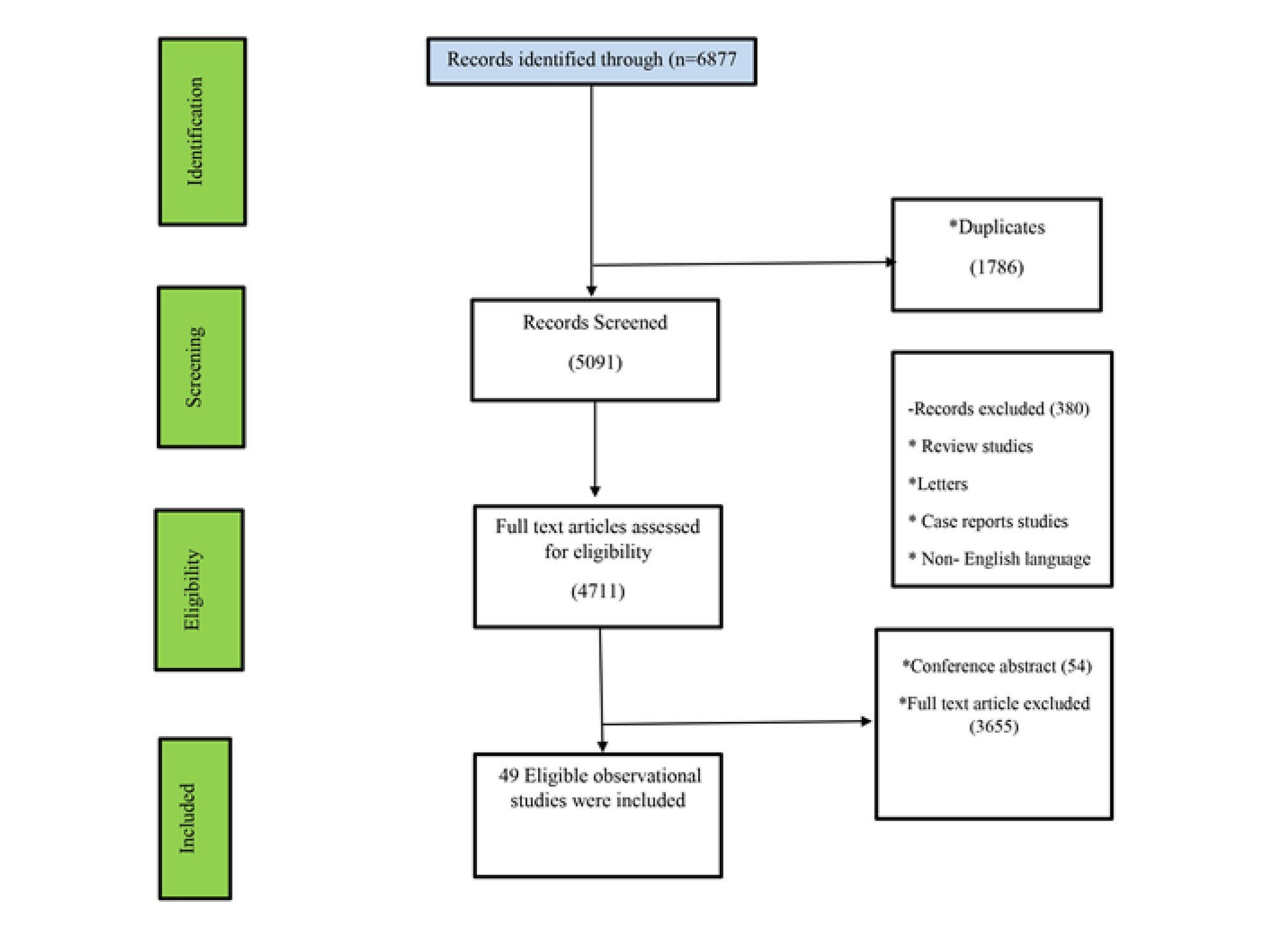
Flowchart of entering studies

**Fig. 2:**
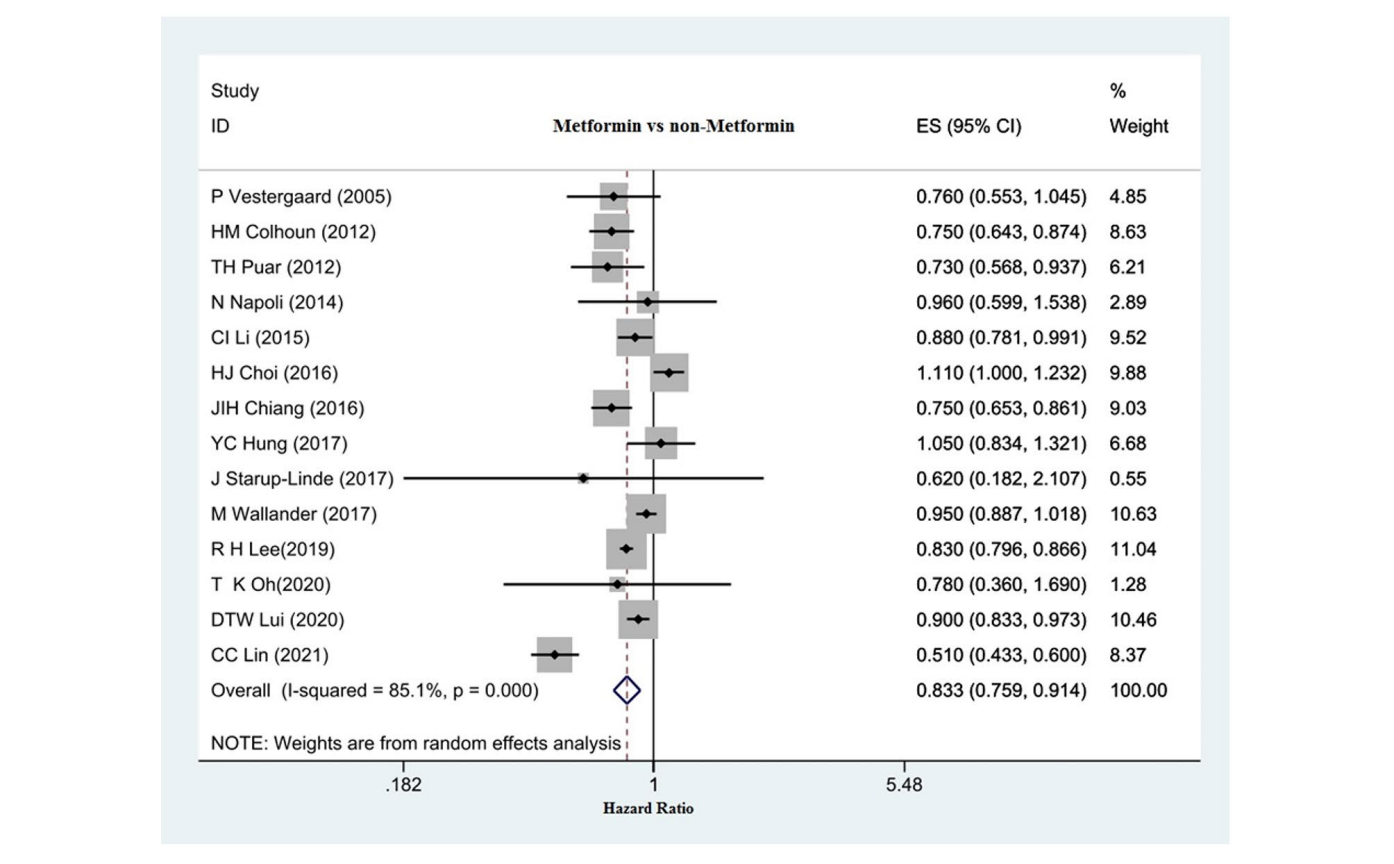
Relationship between metformin use and hip fracture risk in diabetic patients

## Methods

This systematic review and meta-analyses all observational studies which assessed the effect of oral anti-diabetic drugs (metformin and Sulfonylurea) and insulin on hip fractures in patients with type 2 diabetes. The study was conducted as a systematic review and meta-analysis, and it was done based on the checklist of guidelines for conducting systematic review studies (PRISMA). PRISMA diagram was used to show the included and excluded studies.

**Fig. 3:**
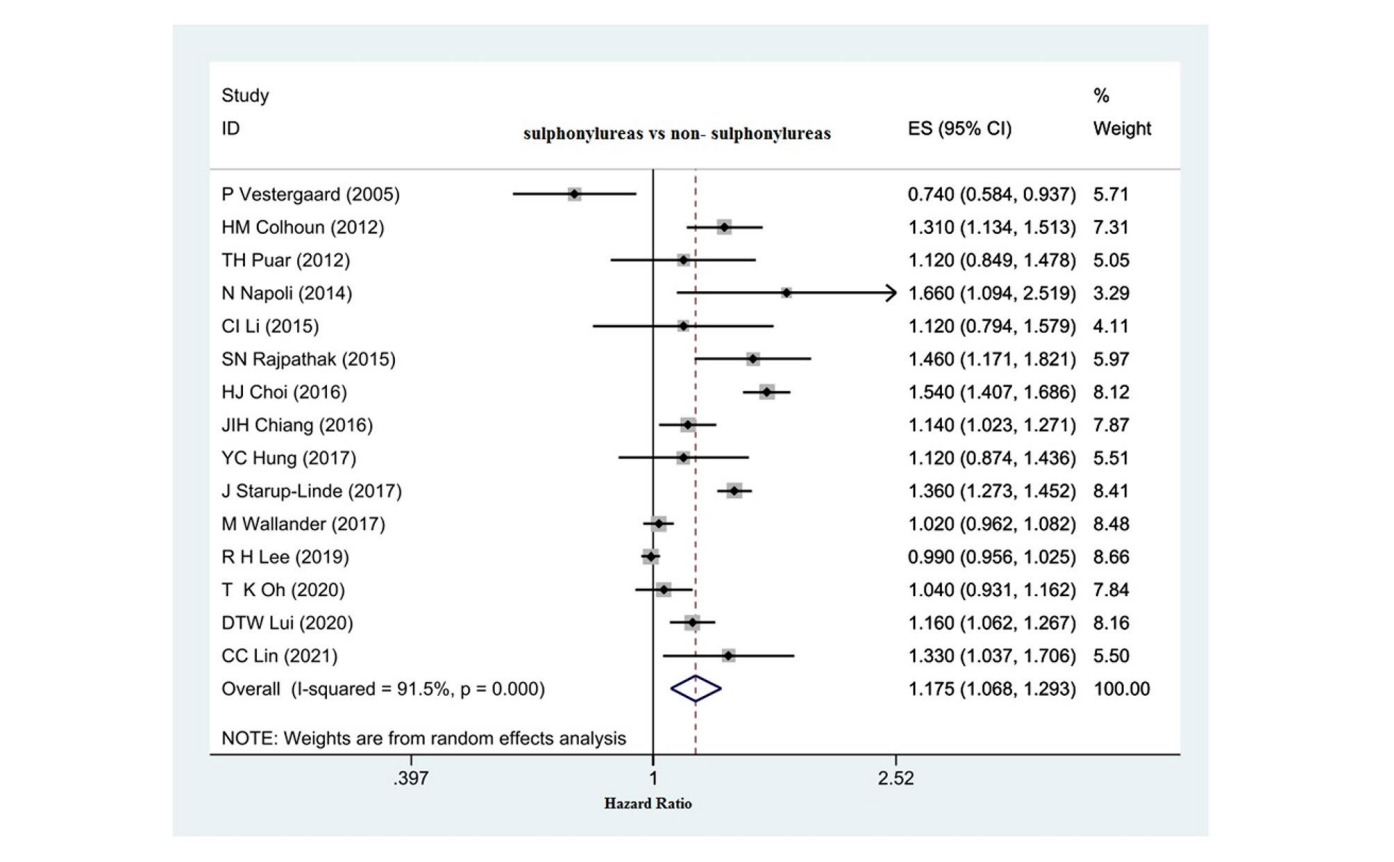
The relationship between the consumption of sulfonylurea compounds and the risk of hip fracture in diabetic patients

### Methods for literature search

After determining the search strategy, databases of PubMed, Scopus, Google Scholar, and Web of Science were searched by two independent researchers (MB) and (ER). The last search was done on September 30, 2022.

The search for sources was limited to human studies, and experimental phase studies were excluded from this meta-analysis. Related studies were searched using keywords and based on PICO. Related keywords were searched in Mesh. The general search strategy for searching for sources was carried out as follows:

)’’ Insulin” OR " Regular Insulin " OR Soluble Insulin’’ OR " Insulin A Chain” OR ‘Sodium Insulin ‘’ OR ‘’ Novolin’’ OR ‘’ Insulin B Chain” OR ‘’Metformin” OR Dimethylbiguanidine” OR Dimethylguanylguanidine* OR " Glucophage” OR ‘Metformin Hydrochloride ‘’ OR ‘’ Metformin HCl’’ OR ‘’ Sulfonylurea Compounds’’ OR ‘’ Hypoglycemic Agents’’ OR ‘’ antidiabetic drugs’’) AND (‘’ Diabetes " OR ‘’Diabetes Mellitus " OR ‘’ Non-Insulin-Dependent Diabetes Mellitus’’ OR " Stable Diabetes Mellitus” OR ‘Diabetes Mellitus, Type II’’ OR ‘NIDDM’’ OR ‘Type 2 Diabetes Mellitus’’ OR ‘’ Adult-Onset Diabetes Mellitus’’ ) and ( ‘’ Hip Fractures’’ OR ‘’ Trochanteric Fractures ‘’ OR ‘’ Intertrochanteric Fractures’’ OR ‘’Subtrochanteric Fractures’’ OR ‘’ Pelvic fracture’’ OR ‘’ Femoral Fractures’’ OR’ ’Fractures ‘’ ).

**Fig. 4:**
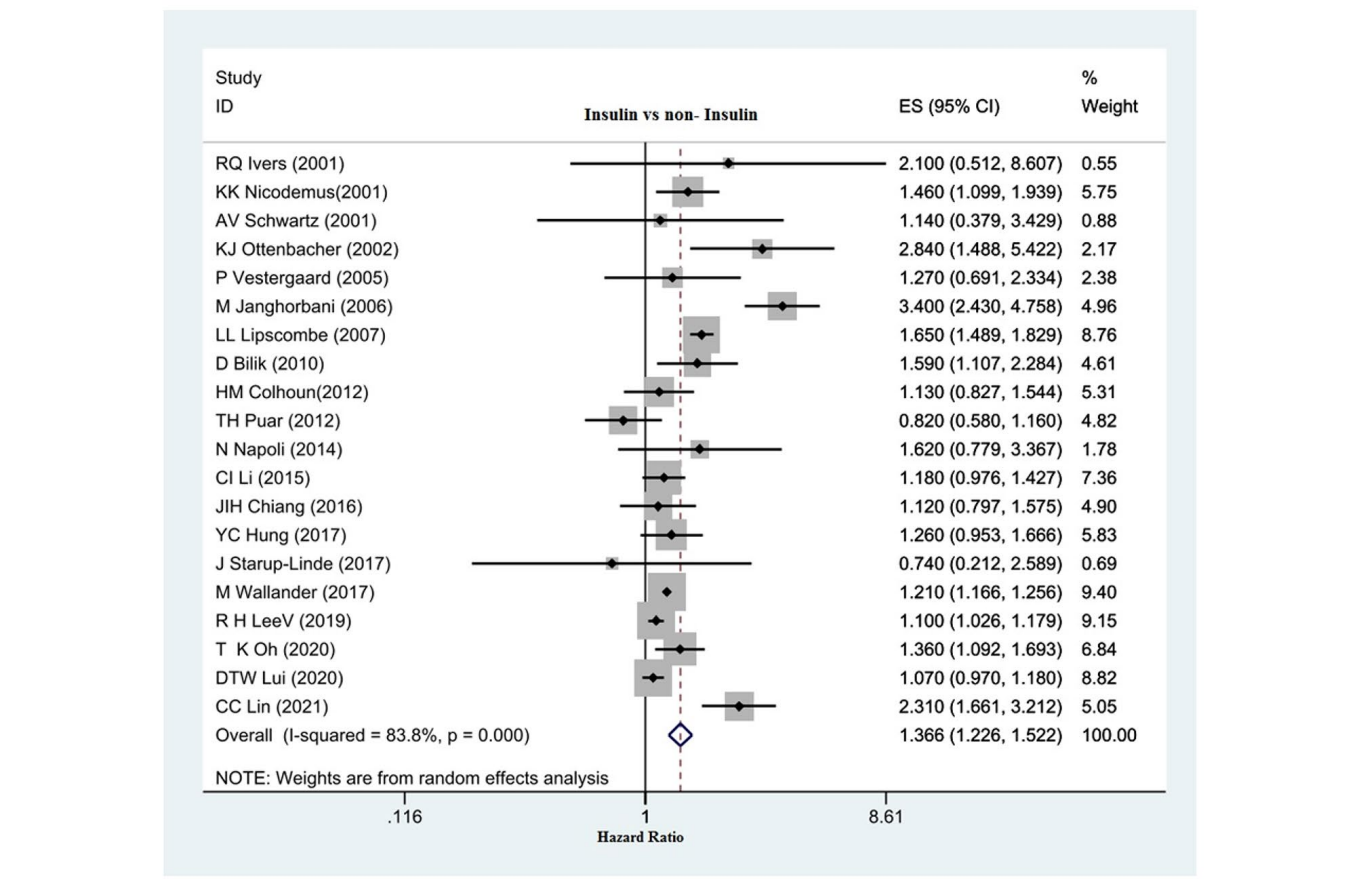
relationship between insulin use and hip fracture risk in diabetic patients

**Table 1:** Characteristics of patients in the studies and the quality of the included studies based on the medication received

Author(Year)	Total subjects	Study design	Total fractures (n)	Hip fractures (n)	Country	Mean Age (Year)	Mean follow-up(year)	Sex(Male)	Mean Duration diabetes (year)	Quality of study
**Metformin**										
P Vestergaard (2005) [8]	162,017	case-control	31,535	10,530	Denmark	66.58	3.43	1,017	NA	Moderate
HM Colhoun (2012) [22]	206,672	prospective Cohort	NA	NA	Scotland	68.5	6.55	NA	8	Moderate
TH Puar (2012) [23]	4,522	case–control	NA	NA	Singapore	77.3	8.8	152	NA	Fair
N Napoli (2014) [24]	5,994	prospective Cohort	450	41	USA	73.08	7.41	241	8.5	Moderate
CI Li (2015) [25]	20,025	retrospective cohort	1514	57	Tiwan	73.6	NA	1142	9.3	Moderate
HJ Choi (2016) [26]	207,558	prospective Cohort	5996	87	South Korea	63.5	9.1	22,118	11.3	Moderate
JIH Chiang (2016) [27]	26,501	prospective Cohort	1217	35	Taiwan	70.22	2.1	11,929	9.16	Good
YC Hung (2017) [28]	7,761	prospective Cohort	2236	195	Tiwan	70.1	3.9	4,424	10.5	Moderate
J Starup-Linde (2017) [29]	180,073	prospective Cohort	5244	1468	Denmark	73.3	8.1	97,239	10.9	Good
M Wallander (2017) [30]	429,313	prospective Cohort	36,132	573	Sweden	80.8	6.7	33,247	NA	Moderate
R H Lee(2019) [31]	662,628	prospective Cohort	130,143	3982	USA	67.55	5.5	3982	NA	Good
T K Oh(2020) [32]	64,878	prospective Cohort	NA	773	South Korea	60.9	4.3	16,422	9.8	Moderate
DTW Lui (2020) [33]	83,282	prospective Cohort	NA	2162	Chinese	70.55	4	39,310	11.8	Good
CC Lin (2021) [34]	105,500	prospective Cohort	2061	67	Taiwan	60.52	3.43	25,275	7.47	Moderate
**Sulphonylureas**										
P Vestergaard (2005) [8]	162,017	case-control	31,535	10,530	Denmark	68.66	8.8	1,271	NA	Moderate
HM Colhoun (2012) [3]	206,672	prospective Cohort	NA	NA	Scotland	66.7	NA	NA	8	Good
TH Puar (2012) [4]	4522	case–control	NA	NA	Singapore	77.1	9.1	154	NA	Moderate
N Napoli (2014) [5]	5,994	prospective Cohort	351	68	USA	73.8	7.41	185	8.5	Moderate
CI Li (2015) [25]	20,025	retrospective cohort	2487	160	Tiwan	73.8	4	1142	9	Good
SN Rajpathak (2015) [35]	42,747	prospective Cohort	383	226	USA	72.5	2.1	7046	NA	Moderate
HJ Choi (2016) [26]	207,558	prospective Cohort	5996	1034	South Korea	65.44	8.12	15,137	9.2	Moderate
JIH Chiang (2016) [7]	26,501	prospective Cohort	1217	114	Taiwan	70.22	3.9	11,929	9.16	Good
YC Hung (2017) [28]	7761	prospective Cohort	514	233	Tiwan	69.88	5.5	4423.77	9.3	Moderate
J Starup-Linde (2017) [29]	180,073	prospective Cohort	5244	2150	Denmark	73.2	1.3	97,239	10.9	Moderate
M Wallander (2017) [10]	429,313	prospective Cohort	36,132	341	Sweden	79.4	3.43	6943	NA	Moderate
R H Lee (2019) [11]	662,628	prospective Cohort	132,957	6796	USA	69.33	6.55	6796	NA	Good
T K Oh (2020) [12]	64,878	prospective Cohort	NA	456	South Korea	65.33	6.7	9,102	NA	fair
DTW Lui (2020) [13]	83,282	prospective Cohort	NA	1693	Chinese	71.33	8.1	39,309	11.7	Moderate
CC Lin (2021) [34]	105,500	prospective Cohort	2061	198	Taiwan	60.51	8.8	25,275	7.47	Moderate
**Insulin**										
RQ Ivers (2001) [36]	4433	prospective Cohort	251	59	Australia	66.2	4.7	1571	NA	Fair
KK Nicodemus(2001) [37]	32,106	prospective Cohort	490	13	USA	62.3	5.6	NA	9.1	Moderate
AV Schwartz (2001) [38]	9654	prospective Cohort	549	6	USA	68.8	9.4	NA	9.2	Fair
KJ Ottenbacher (2002) [39]	3050	prospective Cohort	134	27	USA	71.4	7.5	1213	NA	Fair
P Vestergaard (2005) [2]	162,017	case-control	31,535	10,530	Denmark	69.11	NA	954	NA	Good
M Janghorbani (2006) [40]	109,983	prospective Cohort	1,398	36	NA	61.7	20.4	4154	16.3	Moderate
LL Lipscombe (2007) [41]	142,561	retrospective cohort	NA	58	Canada	68.9	11.2	123,501	NA	Moderate
D Bilik (2010) [42]	180,000	prospective Cohort	786	116	USA	69.9	6.55	1225	NA	Fair
HM Colhoun(2012) [3]	206,672	prospective Cohort	NA	NA	Scotland	69.5	8.8	NA	8	Moderate
TH Puar (2012) [4]	4522	case–control	NA	NA	Singapore	77.5	NA	166	NA	Fair
N Napoli (2014) [5]	5,994	prospective Cohort	80	20	USA	73.5	9.1	43	NA	Moderate
CI Li (2015) [25]	20,025	retrospective cohort	624	55	Tiwan	75.2	7.41	1142	9	Moderate
JIH Chiang (2016) [27]	2650	prospective Cohort	1217	47	Taiwan	70.22	8.12	11,929	9.16	Good
YC Hung (2017) [28]	7761	prospective Cohort	514	86	Tiwan	70	3.9	4423.77	NA	Moderate
J Starup-Linde (2017) [29]	18,073	prospective Cohort	5244	682	Denmark	73.5	5.5	97,240	10.8	Good
M Wallander (2017) [30]	429,313	prospective Cohort	36,132	1119	Sweden	79.3	1.3	18,349	NA	Good
R H Lee (2019) [31]	662,628	prospective Cohort	129,505	3344	USA	65.33	3.43	3344	NA	Good
T K Oh (2020) [32]	64,878	prospective Cohort	NA	165	South Korea	64.88	6.55	768	NA	Moderate
DTW Lui (2020) [33]	83,282	prospective Cohort	NA	472	Chinese	71.54	6.9	39,310	11.3	Good
CC Lin (2021) [34]	105,500	prospective Cohort	2061	85	Taiwan	61.54	8.1	25,275	7.47	Moderate

### Eligibility criteria and data extraction

In this study, until September 30, 2022, we included all retrospective and prospective observational studies that evaluated the relationship between oral anti-diabetic drugs (metformin and sulfonylureas) and insulin with hip fractures, and patients were followed up at least for four years were included. After searching the sources, two independent researchers evaluated the studies using the title and checklist. The study’s eligibility to be included in this meta-analysis was first screened and evaluated by titles, if necessary, by reviewing the abstract. Then, the full texts of the studies that met the inclusion criteria were evaluated to check the inclusion and exclusion criteria. Evaluating the relationship between anti-diabetic medication and hip fractures and studies with a follow-up of at least four years was the criterion for including studies in this systematic review. **Exclusion criteria included**: studies published in a language other than English, case report studies, review articles and meta-analyses, laboratory or animal studies, and lack of access to the full text of the article. After searching in PubMed, Scopus, Web of Sciences, and Google Scholar, 6877 studies were extracted. Endnote version 22 software was used to remove duplicate articles and screen studies. After completing the search, 2166 studies (1786 common and repeated articles among the searched sources, 83 non-English studies, 212 letters to the editor or case reports, and 85 review studies) were removed. The remaining studies (4711) were evaluated regarding the relevance of the titles, purpose, and abstract to the research topic.

After removing 3709 articles, 1002 full texts were studied. Finally, 49 observational studies that assessed the relationship between metformin or insulin or sulfonylurea compound use with hip fractures were included. (Fig. 1).

All needed information to perform a meta-analysis includes the author, year, data sources, type of study design, year, age range, gender distribution, the total number of people examined in each study, the number of diabetic people, the total number of fractures, the number of hip fractures in people who were receiving anti-diabetic drugs, the number of people according to the drugs received, the number of hip fractures based on the type of drug received, the duration of follow-up of patients, the average duration of diabetes in patients, the control group for each drug, BMI, and the risk of hip fracture based on the Hazard ratio index, the confidence interval of 95% and the quality of the studies were extracted. During the data extraction procedure, if there was a difference in a variable in terms of harmony in different studies, the difference was resolved based on the agreement between the two investigators.

### Quality assessment of studies

We applied the observational studies checklists and checked the quality of the studies (The Newcastle-Ottawa Scale (NOS) for Assessing the Quality of Nonrandomized Studies in Meta-Analysis for case-control studies and the Newcastle-Ottawa checklist Quality Assessment Form for Cohort Studies). The evaluation was done by two independent researchers. [13, 14]. If there was a difference between two researchers regarding the quality of a study based on the checklist, this study was also evaluated by a third researcher, and the difference was resolved based the third researcher’s opinion. These checklists evaluate the quality of studies in three sections: Selection, Comparability, and Outcome/Exposure, and give a score for each item. The score range for the cohort and control studies checklist was 0 to 9. The quality classification of the studies includes good (3 or 4 scores for the selection and one or two stars for the comparability and 2 or 3 stars for the outcome/exposure), fair (2 scores for the selection and one or two stars for the comparability and 2 or 3 stars for the outcome/exposure) and poor (0 or 1 score for the selection, 0 stars for the comparability, and 0 or 1 star for the outcome/exposure). The score range for the cohort and control studies checklist was 0 to 9.

**Fig. 5:**
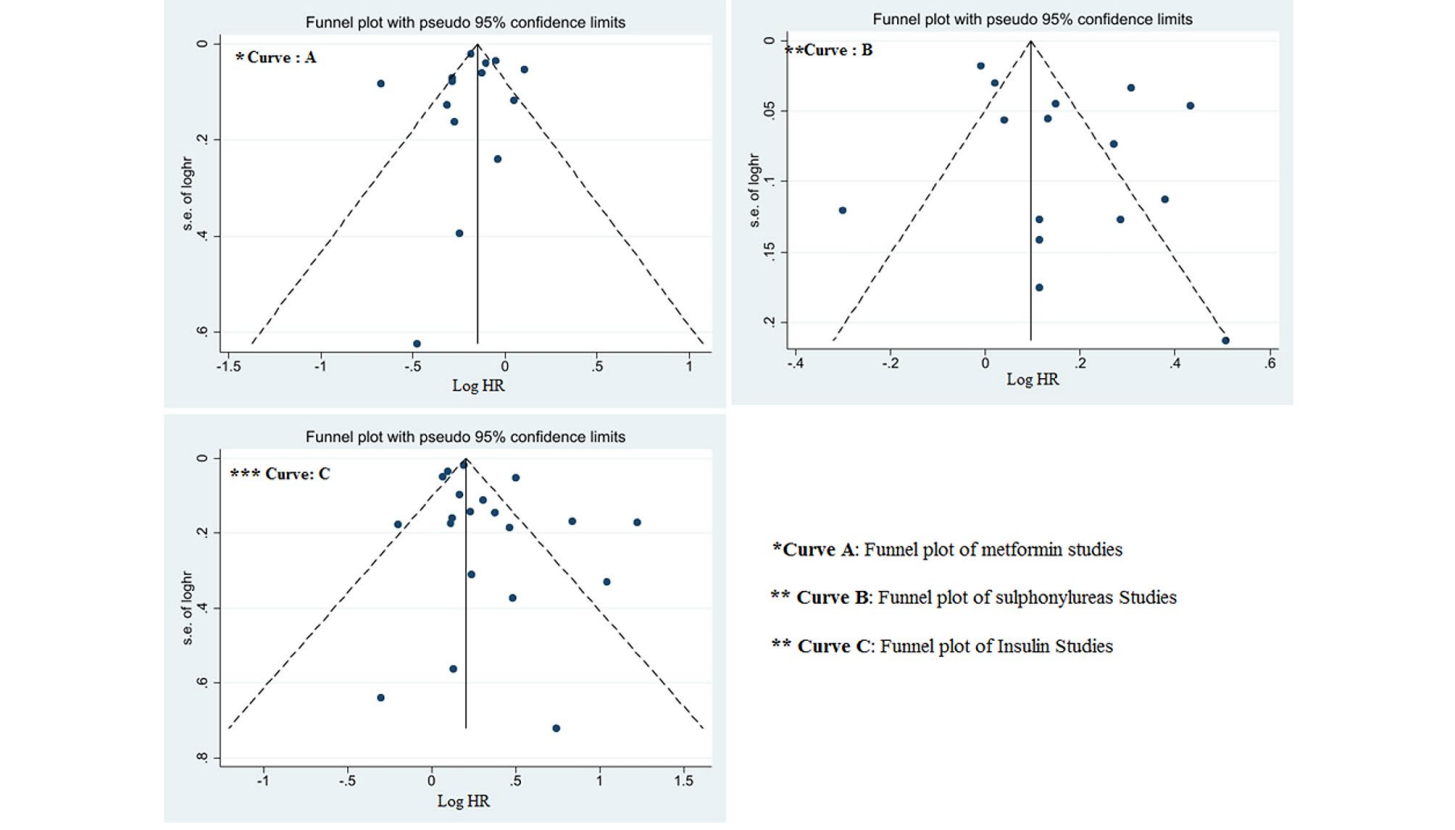
Investigation of publication bias of studies based on received antidiabetic drugs

### Statistical analysis

In all studies, the effect size for survival indicators was extracted using Hazard ratio (HR) and with a confidence interval of 95% (CI 95%). In this review, we investigated the relationship between using metformin, sulfonylureas, and insulin with hip fractures with the adjusted HR index extracted from studies. In almost all cohort studies, the adjusted HR index was used to evaluate the relationship between medication use and hip fracture. If a study used other indices such as RR or OR for case-control studies, these indices were converted into HR using the number of events in each treatment group. The random effect method was used to control the effects of the sample size of the studies to estimate the final effect size for each index. In this section, based on the variances of each study, the weight of each study was initially calculated based on the Fix Effect model as the inverse of the variance. Then, having the obtained prevalence values, the weight of each study was calculated using techniques that were combined to determine heterogeneity within and between studies (Random) and Dersiminian and Laird. Cochran’s Q and I2 tests were used to assess heterogeneity between studies. Publication bias between studies was assessed for each drug using Egger’s test. Due to the absence of publication bias, there was no need to use trim and fill analysis to solve the publication bias for the relationship between the risk of fracture with any of metformin, sulfonylureas, and insulin in different studies. The pooled HR with a 95% confidence interval was used to estimate the outcome of the overall relationship between hip fracture and anti-diabetic drugs. Stata 17.0 software was used to experiment and analyze the data of the studies.

## Results

In general, 49 studies, including 6,631,297 participants, were assessed in this meta-analysis. A total of 14 studies, including 2,166,724 participants assessed the relationship between metformin use and hip fracture risk. The median age of the participants was 69.75 years. A total of 12,371 hip fractures were observed in 385,761 patients taking metformin, and the overall incidence of hip fractures in these patients was reported as 3.21%. The follow-up period of the patients was 5.89 years. A total of 2,209,471 studies evaluated the relationship between the use of sulfonyl compounds and the risk of hip fracture. A total of 13,999 cases of hip fracture were observed in patients taking these medicinal compounds. The incidence of hip fracture in these patients was reported to be 4.48%. The mean follow-up period in these patients was reported to be 5.77 years.

Twenty studies with 2,255,102 patients investigated the relationship between long-term insulin use and the risk of hip fracture; based on the results of hip fracture incidence in 155,866 patients who used insulin and a fracture incidence of 10.86% was estimated. The main age and mean duration of follow-up in these patients were 67.58 and 7.63 years, respectively. According to the study evaluation checklist, most studies were of good quality. Study characteristics are reported separately in Table 1 for each treatment received.

### The relationship between metformin use and hip fracture risk

Fourteen studies (13 cohort studies and one case-control study) investigated the relationship between metformin use and hip fracture risk. The pooled effect showed that metformin was significantly associated with a reduced risk of hip fracture compared to diabetic patients who did not take metformin. (HR: 0.833, 95% CI: 0.759, 0.914, P: 0.001) (Fig. 2). No significant relationship was reported for the effect of bias on the overall outcome of the studies that investigated the relationship between metformin and the risk of hip fracture. (Egger test: -0.59, p: 0.61, 95%CI: -3.11,1.88). (Fig. 5-A)

### Relationship between sulfonylurea consumption and hip fracture risk

Fifteen studies (13 cohorts and 2 case-control) investigated the relationship between sulfonylureas and hip fracture risk. The pooled effect showed that the consumption of sulfonylurea compounds was significantly associated with an increased risk of hip fracture. (HR: 1.175, 95% CI: 1.068, 1.293, P: 0.001) (Fig. 3) that according to Egger’s test results, no publication bias was reported in these studies. (Egger test: 2.03, p: 0.17, 95% CI: -1.01, 2.53).(Fig. 5-B).

### The relationship between insulin use and hip fracture risk

Overall, 20 studies (18 cohort studies and two case-control studies) investigated the relationship between insulin use over 12 months and hip fracture risk. According to the pooled results, the risk of hip fracture in patients receiving insulin was significantly higher than in diabetic patients who did not receive insulin. (HR: 1.366, 95% CI: 1.226, 1.522, P: 0.001). (Fig. 4) A significant relationship for the effect of publication bias on the overall outcome according to Egger’s test for studies that assessed the relationship between insulin use and the risk of hip fracture was not observed Egger test: 1.047, p: 0.156, 95% CI: -0.43,2.53) Diagram (Fig. 5-c).

## Discussion

So far, meta-analysis studies have been conducted regarding the effect of diabetes and its drugs on fractures. Still, according to our knowledge, no meta-analysis study has been conducted on the relationship between diabetes drugs and hip fractures. Therefore, in this study, for the first time, the effect of diabetes drugs metformin, sulfonylureas, and insulin on the risk of hip fracture was investigated in a meta-analysis. Our results show that the use of metformin was significantly associated with a reduction in the risk of hip fracture compared to diabetic patients who did not use metformin (HR: 0.833, 95% CI: 0.759, 0.914, P: 0.001). A meta-analysis study conducted by Salari-Moghaddam et al. [15] to investigate the effect of metformin use on total fractures showed that the use of metformin significantly reduces the risk of fractures in patients with diabetes. Their results were in line with the results of our study. The mechanism of metformin action on bone is through activating AMP-activated protein kinase (AMPK). AMPK may directly affect bone turnover by increasing osteoblastogenesis and decreasing osteoclastogenesis. Metformin also increases osteogenesis by activating AMPK and fructose 1,6-biphosphate pathways. [16, 17].On the other hand, various studies have shown that metformin activates the differentiation of mesenchymal stem cells towards osteoblasts and inhibits osteoclast differentiation [18]. In addition, metformin prevents fat production in the bone marrow by reducing endothelial nitric oxide synthase (eNOS).

### The effect of sulfonylureas on hip fracture

Also, our study shows that the consumption of sulfonylurea compounds is significantly associated with an increased risk of hip fracture. In the meta-analysis study by Zhen Zhang et al. [19], the rate of fractures (total fractures) in patients using sulfonylureas was higher than in the other group, and it showed that the use of sulfonylureas significantly increased the probability of fractures in patients with diabetes. Glimepiride belonging to sulfonylureas, plays an important role in stimulating bone formation. Glimepiride prevents bone loss associated with menopause but has no role in bone metabolism. However, some studies have shown that using sulfonylureas as an anti-diabetic drug in elderly diabetic patients increases the risk of bone fracture [20].

Another part of our study investigated the relationship between insulin use and hip fracture. This study showed that according to the pooled effects, the risk of hip fracture in patients who received insulin was significantly higher than in diabetic patients who did not receive insulin. (HR: 1.366, 95% CI: 1.226, 1.522, P: 0.001). These results were in line with Yuxian Zhang et al.‘s study[21], which investigated the relationship between insulin use and fractures in patients with diabetes in a meta-analysis study. Their results also showed that the use of insulin significantly increases the risk of fracture. Regarding the mechanism of insulin, similar to sulfonylureas, the clinical effect of insulin on bone mainly results from the higher incidence of hypoglycemia. It is associated with the risk of falls and bone fractures [20].

Our study has limitations and strengths that need to be mentioned:

#### The limitations

(1) this study is the result of the analysis of different studies in different regions and populations, and these differences can affect the overall effect. (2) In these studies, the use of drugs was self-reported, which can affect the final effect.

Strengths: (1) This study, for the first time, investigated the relationship between three diabetic drugs, insulin, metformin, and sulfonylureas, with hip fracture as a meta-analysis. (2) According to the study quality checklist, most studies included in our meta-analysis were of high quality.

## Conclusion

The results of this study are showed that taking metformin reduces the risk of hip fracture, and insulin and sulfonylurea increase the risk of hip fracture. This study’s results can guide prescription and treatment for diabetic patients, especially at older ages.

## Data Availability

The datasets used and/or analysed during the current study are available from the corresponding author on reasonable request.

## Abbreviations

HF: Hip Fracture
AMPK: AMP-activated protein kinase
HR: Hazard ratio
95% CI: confidence interval of 95%
